# Tree Species Richness and Neighborhood Effects on Ectomycorrhizal Fungal Richness and Community Structure in Boreal Forest

**DOI:** 10.3389/fmicb.2021.567961

**Published:** 2021-02-22

**Authors:** Eveli Otsing, Sten Anslan, Elia Ambrosio, Julia Koricheva, Leho Tedersoo

**Affiliations:** ^1^Institute of Ecology and Earth Sciences, University of Tartu, Tartu, Estonia; ^2^Department of Biological Sciences, Royal Holloway University of London, Egham, United Kingdom; ^3^Natural History Museum, University of Tartu, Tartu, Estonia

**Keywords:** ectomycorrhizal fungi, fungal richness, neighborhood effects, boreal forest, fungal community structure

## Abstract

Tree species identity is one of the key factors driving ectomycorrhizal (EcM) fungal richness and community composition in boreal and temperate forest ecosystems, but little is known about the influence of tree species combinations and their neighborhood effects on EcM communities. To advance our understanding of host plant effects on EcM fungi, the roots of silver birch, Scots pine, and Norway spruce were analyzed using high-throughput sequencing across mature boreal forest exploratory plots of monocultures and two- and three-species mixtures in Finland. Our analyses revealed that tree species identity was an important determinant of EcM fungal community composition, but tree species richness had no significant influence on EcM fungal richness and community composition. We found that EcM fungal community composition associated with spruce depends on neighboring tree species. Our study suggests that at a regional-scale tree species identity is the primary factor determining community composition of root-associated EcM fungi alongside with tree species composition effects on EcM fungal community of spruce in mixed stands.

## Introduction

Effects of aboveground diversity on belowground diversity and *vice versa* are context-dependent and difficult to predict (Wardle et al., 2004). Plant diversity affects soil microbes through host plant density (Peay et al., 2011), identity (Tedersoo et al., 2010; Nguyen et al., 2016b; Nagati et al., 2018; Chen et al., 2019), richness (Kernaghan et al., 2003; Ishida et al., 2007; Tedersoo et al., 2016), phylogenetic diversity (Ishida et al., 2007; Gao et al., 2013; Tedersoo et al., 2013) and litter quality (Conn and Dighton, 2000; Aponte et al., 2010, 2013). Microbial interactions can drive ecosystem functions, such as plant biodiversity, productivity, and variability as shown by van der Heijden et al. (1998, 2008) based on arbuscular mycorrhizal fungal communities. It is known that plant diversity and productivity increase with increasing diversity of ectomycorrhizal (EcM) fungi (Baxter and Dighton, 2001; Jonsson et al., 2001; Kernaghan et al., 2003). EcM fungal communities have a major part in driving forest soil processes, such as soil organic matter decomposition, nutrient cycling, and carbon (C) sequestration (Read and Perez-Moreno, 2003; Clemmensen et al., 2015; Lindahl and Tunlid, 2015). Plant-fungus interactions play an important role in ecosystem functioning, but plant diversity effects on microbial communities in forest ecosystems are not well-understood.

One of the most challenging issues in above- and belowground relationships is to understand the extent to which plant species diversity influences fungal diversity and *vice versa* (Allen et al., 1995). It has been suggested that plant species richness should have positive effect on soil microbial richness due to greater environmental heterogeneity (Bruns, 1995; Wardle, 2006; Dickie, 2007). Each plant species provides consistent patterns in the chemical composition of litter and root exudates, which shape microbial community through affecting the characteristics of the rhizosphere microenvironment (Gobran et al., 1998; Aponte et al., 2010; Waring et al., 2015; Zhalnina et al., 2018). Accordingly, positive correlation between plant and soil fungal (including EcM fungi) diversity has been observed at local scale in several studies (Kernaghan et al., 2003; Dickie, 2007; Tedersoo et al., 2016). In contrast, Nguyen et al. (2016b) reported no significant effect of increasing plant species richness on EcM fungal species richness at the local scale. Furthermore, Tedersoo et al. (2012, 2014a) concluded that EcM fungal richness is not dependent on plant species richness at the global scale.

Tree identity of plant species has been demonstrated to strongly affect both species richness and community composition of EcM fungi at local and global scales (Dickie, 2007; Ishida et al., 2007; Tedersoo et al., 2012; Bogar and Kennedy, 2013). The influence of plant identity on EcM fungi is associated with plant phylogeny. Phylogenetic relatedness among host plants can predict species richness and community composition of EcM fungi (Ishida et al., 2007; Põlme et al., 2013; Tedersoo et al., 2013). Stands with more phylogenetically distant plants harbor higher richness of fungi and hold more dissimilar fungal community composition in comparison to stands with closely related host species (Smith et al., 2009; Nguyen et al., 2016b). It is suggested that the influence of plant phylogenetic diversity on EcM fungal richness is most evident at higher plant taxonomic levels; for example in mixtures of gymnosperm and angiosperm hosts (Nguyen et al., 2016b). Furthermore, positive relationships between plant and EcM fungal species diversity can be explained by the presence of EcM fungal specialists in mixtures of coniferous and broadleaf tree species (Kernaghan et al., 2003; Ishida et al., 2007). Although some EcM fungi show specificity and/or preference to certain plant genera, most common EcM fungi can colonize a broad range of hosts (Molina et al., 1992; Molina and Horton, 2015). In mixed-species stands, root-colonizing fungi with low host specificity and/or preference can be shared among different tree species (Simard et al., 1997; Horton and Bruns, 1998). Therefore, in order to characterize the structure of EcM fungal communities, it is important to consider the potential impact of stand composition.

Tree species composition may affect EcM fungal richness and community composition in focal trees through neighborhood effect (Haskins and Gehring, 2004; Bahram et al., 2011; Jairus et al., 2011; Bogar and Kennedy, 2013; Santolamazza-Carbone et al., 2019). Neighboring plants of different species can create community shifts compared with monocultures through different mechanisms. In Bogar and Kennedy (2013), priority effects among *Alnus*- and *Betula*-associated EcM fungi allowed the established *Alnus* to control the structure of the *Betula* community. *Alnus*, the preferred host, may have provided sufficient photosynthate to facilitate colonization of *Betula*, which the fungi typically would not associate (Bogar and Kennedy, 2013). Kohout et al. (2011) found that changes in EcM fungal communities of pine species (*Pinus strobus* and *Pinus sylvestris*) might have been caused by the selective stimulation/inhibition effects of some ericaceous dwarf shrubs (*Vaccinium myrtillus* and *Vaccinium vitis-idaea*) or a preference of some mycobionts for ericaceous to coniferous roots. Also, the influence of host neighborhood can be associated with the unique properties created by different tree species that are not found in single-species stands (Cavard et al., 2011). Differences in litter quality, soil nutrients, temperature, and moisture (Brandtberg et al., 2000; Légaré et al., 2005) can alter EcM fungal communities (Conn and Dighton, 2000; Aponte et al., 2010, 2013). In mixed stands, different tree species create spatial heterogeneity in the soil environment that lead to diversity of microhabitats and suitable conditions for a greater variety of EcM fungal species (Conn and Dighton, 2000).

Here, we aimed to characterize the effects of tree species richness and composition of neighbouring trees on EcM fungal community composition and richness in monoculture and mixed forest stands. We hypothesized that in boreal ecosystem (i) EcM richness increases with increasing number of tree species; (ii) tree species identity affects EcM fungal richness and community composition; (iii) EcM fungal richness and community composition are affected by tree neighborhood context. EcM fungal diversity of silver birch, scots pine, and Norway spruce, from monocultures and two- and three-species mixed plots were characterized by Illumina MiSeq sequencing.

## Materials and Methods

### Study Site and Sampling

Root samples were collected from the exploratory sites established as part of the FunDivEUROPE EU project in North Karelia, Finland (62.6°N, 29.9°E).^1^ FunDivEurope exploratory platform has been established to assess tree species diversity effects on forest ecosystem functioning in mature European forests. The exploratory platform includes mature forest plots along tree species diversity gradients in six major European forest types. Within each site, focus is on regionally common tree species combinations from monocultures to mixed species stands. Special attention in site selection was given to community evenness and avoiding covariation with other environmental factors (e.g., soil variation and topography). The exploratory sites were established to complement the existing networks of experimental research sites and inventories (Baeten et al., 2013). The Finnish exploratory platform is composed of 28 plots (30 × 30 m) with monocultures, two- and three-species mixtures (1-, 2-, and 3-species richness levels) of silver birch (*Betula pendula* Roth.), Scots pine (*Pinus sylvestris* L.), and Norway spruce (*Picea abies* L. Karst.). In some instances, downy birch (*Betula pubescens* Ehrh.) has self-established by seed. The 28 plots of the exploratory region were located in an area of 150 km by 150 km. The age of forest plots ranged from 36 to 53 years at the time of sampling. There are four replicate plots of each of the tree species combinations (birch, pine, spruce, birch-pine, birch-spruce, pine-spruce, and birch-pine-spruce). In the exploratory site, the mean annual temperature is 2.1°C and the mean annual precipitation is 700 mm. The plots were situated at altitude between 80 and 200 m above sea level. The soils are Podzols above mica schist bedrock and granites and granodiorites bedrock.

In August–September 2015, samples were collected from 28 plots by sampling fine roots from four trees per species per plot (i.e., four in monocultures, eight in two-species mixtures, and 12 in three-species mixtures). Four replicate plots of each of the species composition were sampled, resulting in 192 samples. Coarse roots were traced to 10–20 cm from the tree trunk. Smaller root branches attached to these were dug out, with 15 cm root length including most intact fine roots and mycorrhizas, which were cut-off and placed into plastic bag. Some fine soil was added to keep the sample moist. Samples were stored in a refrigerator at about 4°C until processing. In the laboratory, attempting to prevent detachment and loss of mycorrhizas, roots were carefully cleaned from adhering soil under running tap water. Each mycorrhizal root sample was cut to equal length fragments. From each sample, 10 fragments were randomly selected and washed again with tap water. These root samples were placed on a tissue paper to remove the excess water and then placed into tubes containing CTAB buffer [1% cetyltrimethylammonium bromide, 100 mM Tris-HCl (pH 8.0), 1.4 M NaCl, and 20 mM EDTA] for further molecular analyses.

### Molecular Analysis

Before DNA extraction, root samples were washed from CTAB buffer and then homogenized in 2-ml Eppendorf tube using two 3-mm tungsten carbide beads in Mixer Mill MM400 (Retsch GmbH, Haan, Germany) at 30 Hz for 5 min. The PowerSoil DNA Isolation Kit (MoBio, Carlsbad, CA, United States) was used to extract DNA from homogenized root samples following the manufacturer’s protocols. PCR was carried out using a mixture of five forward primers ITS3ngsMixTag1-5 (CTAGACTCGTCAHCGATGAAGAACGYRG) in equimolar concentration and a degenerate reverse primer ITS4ngs (TCCTSCGCTTATTGATATGC; Tedersoo et al., 2014b). The ITS4ngs primer was supplemented with unique 10–12 base pairs long tags per sample (Supplementary Table S1). Tags were modified from those recommended by Roche (Basel, Switzerland) to differ by >3 bases, to start only with adenosine and to comprise similar proportions of adenosine and thymidine (between 30 and 70%) to equalize their affinities in an adapter ligation step (Tedersoo et al., 2014b). The PCR mixture comprised 0.6 μl template DNA, 0.5 μl each of the primers (20 μM), 5 μl 5 × HOT FIREPol Blend Master Mix (Solis Biodyne, Tartu, Estonia), and 13.4 μl double-distilled water. PCR was carried out in two replicates in the following thermocycling conditions: an initial 15 min at 95°C, followed by 30 cycles of 95°C for 30 s, 55°C for 30 s, 72°C for 1 min, and a final cycle of 10 min at 72°C. PCR products (typically 350–400 bp) from replicate samples were pooled and their relative quantity was estimated by running 5 μl DNA on 1% agarose gel for 25 min. DNA samples with no visible bands were re-amplified with 35 cycles and DNA samples with strong bands were re-amplified with 25 cycles. Both negative and positive controls were included in PCR and sequencing runs. PCR products were pooled at approximately equimolar ratio as determined by gel band strength. Samples were combined into two libraries that were purified by FavorPrep™ Gel/PCR Purification Kit (Favorgen-Biotech Corp., Austria), following the manufacturer’s instructions. DNA from each library was quantified using Qubit® 2.0 Fluorometer (Invitrogen, Life Technologies, CA, United States) and dsDNA High Sensitivity assay kit (ThermoFisher Scientific, Waltham, United States). Amplicons were pooled into two libraries and subjected to adaptor ligation and Illumina MiSeq sequencing (2 × 300 paired-end) in NERC Biomolecular Analysis Facility (Liverpool, United Kingdom).

### Bioinformatic Analysis

Illumina MiSeq sequencing resulted in 3,038,778 raw reads that were processed using PipeCraft platform (v1.0; Anslan et al., 2017). Paired-end reads were merged and quality-trimmed using VSEARCH v1.1.11 (Rognes et al., 2016), with maximum expected error rate of 1. The 2,810,505 filtered sequences were re-assigned to samples based on the tags using mothur v1.36.1 (Schloss et al., 2009) by allowing one mismatch to tag sequence. Chimera filtering of the sequences was performed using VSEARCH based on UNITE v6 reference database (Abarenkov et al., 2010b) and *de novo* option. To extract the full-length ITS2 subregion for clustering purpose, reads were processed with ITSx 1.0.9 (Bengtsson-Palme et al., 2013) to remove flanking gene fragments. Full-length ITS2 reads were assigned to operational taxonomic units (OTUs) by clustering at 97% similarity threshold with CD-Hit v4.6 (Fu et al., 2012). All OTUs represented by a single sequence (singletons) were removed. The most abundant sequence of each cluster was selected as a representative for BLASTn sequence similarity search (word size = 7; gap open = 1; gap extension = 2; reward = 1; penalty = −1) against both International Nucleotide Sequence Databases Collaboration (INSDc) and UNITE. Also, BLASTn searches were run against reference sequences of fungi in 99.0% similarity species hypothesis (SH) that include third-party taxonomic and metadata updates (Kõljalg et al., 2013; Nilsson et al., 2014) as implemented in the PlutoF workbench (Abarenkov et al., 2010a).

We used BLASTn output values (similarity percentage, e-value, and match length/sequence length ratio) of taxonomic assignment to remove remaining potential artefacts and positive and negative controls to account for tag switching errors and contaminants. We considered 10 best-matching references for each OTU to annotate taxa as accurately as possible, and we ran manual BLASTn searches against the INSDc if no reliable taxonomy was revealed. BLASTn *e*-values < e^−50^ were considered reliable to assign OTUs to kingdoms, whereas OTUs with e-values > e^−20^ were treated as “unknown” taxa and removed from the analyses. *E*-values between e^−20^ and e^−50^ were manually checked against the 10 best matches for accurate assignment. OTUs with sequence length < 250 base pairs (over the entire amplicon length) and match/sequence length < 70% were excluded as potential artefacts. Based on sequence distribution in negative and positive controls, we excluded OTU sequence counts = 1 per sample to remove most of the potential tag-switching errors. We relied on 98, 90, 85, 80, and 75% sequence identity as a criterion for assigning OTUs to species, genus, family, order, or class level, respectively (Tedersoo et al., 2014b). Each fungal genus, family or order was assigned to functional categories based on FUNGuild (Nguyen et al., 2016a). Taxa were considered to be EcM if they matched to any sequences belonging to EcM fungal lineages and exhibited sequence blast score/sequence length above the predetermined lineage-specific thresholds (Tedersoo and Smith, 2017). Annotated sequence data and detailed metadata are given in Supplementary Tables S2 and S3, respectively.

### Statistical Analysis

We used the phyloseq package (McMurdie and Holmes, 2013) in R for visualizing taxonomic assignments of EcM fungi at genus level. Venn diagrams for graphical descriptions of unique and shared EcM fungal OTUs between different tree species and tree species composition plots were calculated using the VennDiagram package in R (Chen, 2018). For richness modelling of EcM fungi, we calculated the standardized residuals of the number of OTUs in relation to the square-root of the number of obtained sequences to account for differences in sequencing depth (Tedersoo et al., 2014b). Standardized residuals were obtained from regression analyses performed in STATISTICA 12 (StatSoft Inc., Tulsa, OK, United States). We used the vegan package (Oksanen et al., 2017) in R to calculate evenness index. Sample evenness was determined by dividing the exponential of Shannon-Wiener index by the number of OTUs (Gardener, 2014). In richness, analyses of EcM fungi, tree species richness and composition were considered as fixed factors, while tree species identity (nested in plot) and plot were included as random factors. GLM models (Type I SS) were used to test the effects of plot, tree species richness, composition, and identity on standardized residuals of OTU richness and evenness as implemented in STATISTICA. Using one-way and two-way ANOVA, we performed Tukey’s *post hoc* tests to distinguish statistically significantly different groups, using the R package agricolae (De Mendiburu, 2014).

PRIMER v6 (Clarke and Gorley, 2006), permutational multivariate analysis of variance (PERMANOVA; Anderson et al., 2008) with 999 permutations (Type I SS) was used to test the effect of tree species identity, richness and composition, and plot on EcM community composition in root samples. The OTU matrix (including OTUs with frequency > 1 across data set) was subjected to Hellinger transformation using vegan package in R prior to community analysis. We applied the Bray-Curtis metric for the transformed sequence abundance data. Tree species richness and composition were considered as fixed factors, while tree species identity (nested in plot) and plot were included as random factors. Statistical significance level was considered at *α* = 0.05. To visualize the effect of tree species identity, richness, and composition, non-metric multidimensional scaling (NMDS) was performed on Hellinger-transformed abundance data using the function metaMDS in the vegan package with default settings.

## Results

### Identification of EcM Fungi

**Figure 1:**
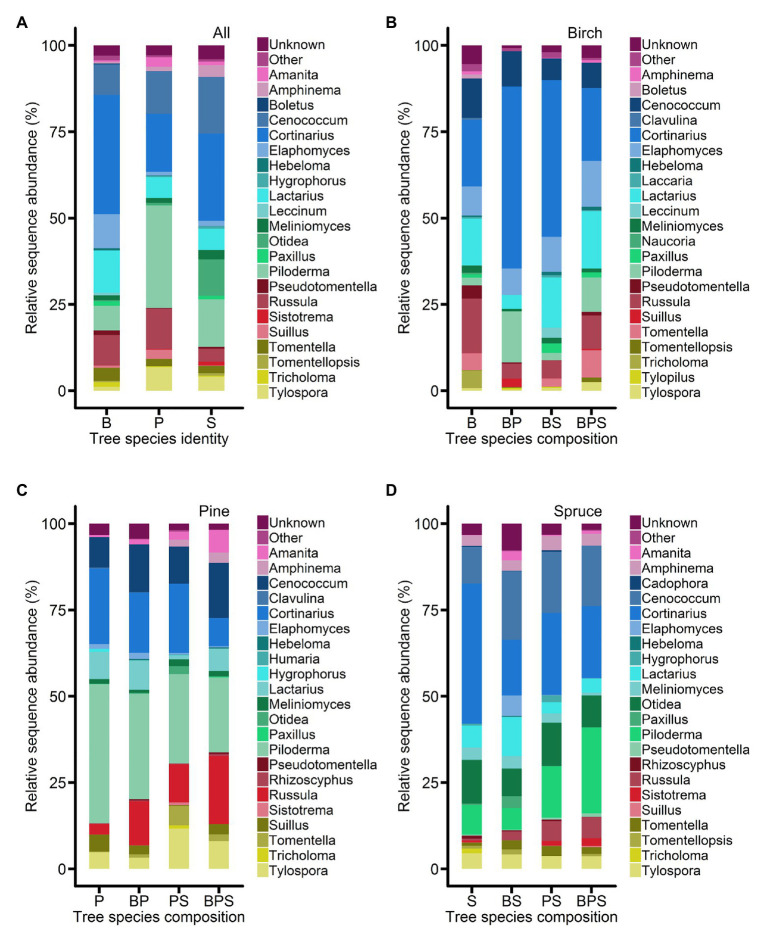
Abundances of major ectomycorrhizal (EcM) fungal genera **(A)** on the roots of *Betula pendula*, *Pinus sylvestris*, and *Picea abies*. Abundances of major EcM fungal genera on the roots of **(B)** *Betula pendula*, **(C)** *Pinus sylvestris*, and **(D)** *Picea abies* in different forest stands (monoculture and mixed stands). The data represent the mean values of the relative abundances of ITS2 amplicons expressed as percentages. B, birch; P, pine; S, spruce; BP, birch-pine; BS, birch-spruce; PS, pine-spruce; and BPS, birch-pine-spruce.

Altogether, 2,079,719 high-quality sequences from 191 (one sample failed to sequence) samples were clustered to 5,739 OTUs, of which 5,489 OTUs were considered to be fungi. Based on sequence matches and predetermined lineage-specific thresholds to existing EcM fungal lineages, we detected 939,923 sequences and 1,428 OTUs of EcM fungi from root samples. Agaricales (274,897 reads), Atheliales (231,290 reads), Hysteriales (131,651 reads), and Russulales (113,397 reads) were dominant orders, whereas *Cortinarius* (242,586 reads), *Piloderma* (160,369 reads), *Cenococcum* (131,651 reads), and *Lactarius* (60,416 reads) were dominant genera. At fungal genus level, the composition of EcM mycobionts of birch, pine, and spruce marginally varied among stands of different tree species composition (Figure 1). On birch, *Cortinarius* was relatively more abundant in birch-pine and birch-spruce mixtures compared to birch-pine-spruce mixture and birch monoculture, whereas on spruce, *Cortinarius* was relatively more abundant in monoculture compared to the mixtures. *Piloderma* was relatively more abundant in pine root samples. On birch, *Piloderma* was relatively more abundant in birch-pine and birch-pine-spruce mixtures, and on spruce, *Piloderma* was relatively more abundant in pine-spruce and birch-pine-spruce mixtures (Figure 1).

**Figure 2:**
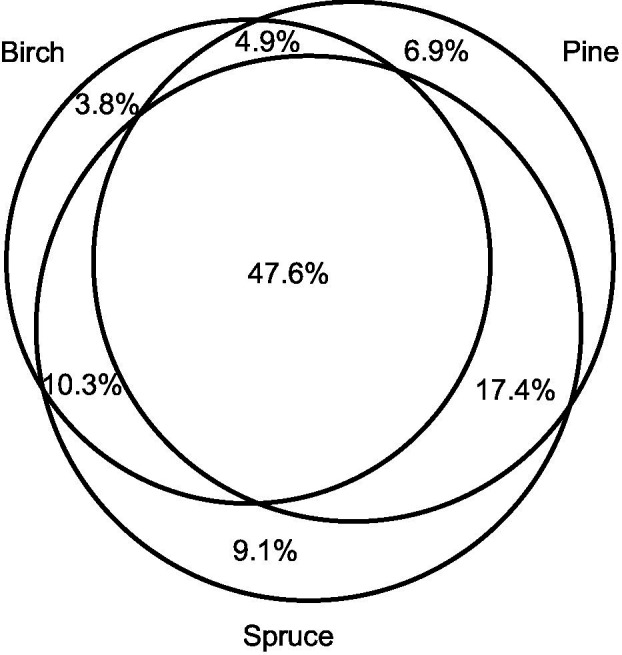
A Venn diagram displaying the proportion of EcM fungal operational taxonomic units (OTUs) shared among *Betula pendula*, *Pinus sylvestris*, and *Picea abies*.

Birch, pine, and spruce harbored 773, 894, and 993 EcM fungal OTUs, respectively. Of the 965 non-singleton OTUs, 19.8% (192 OTUs) occurred on a single host species, whereas 47.6% (459 OTUs) were shared among all tree species (Figure 2). The single-host OTUs accounted for only 2.2% of sequences. Spruce exhibited the highest proportion of unique OTUs (9.1%, 88 OTUs) and proportion of sequences (1.4%). Of the pairwise shared OTUs, largest proportion was shared between pine and spruce (65.0%, 627 OTUs), followed by birch and spruce (57.9%, 558 OTUs), and birch and pine (52.5%, 506 OTUs). The proportions of shared fungal OTUs among different tree species combinations were visualized separately for birch, pine, and spruce samples (Supplementary Figures S1A–C). About 22.1% of birch (142 OTUs), 18.6% of pine (138 OTUs), and 25.8% of spruce fungal OTUs (210 OTUs) were shared among all tree species combinations. Spruce harboured the highest proportion of unique OTUs in birch-spruce mixture (8.7%, 71 OTUs). Spruce monoculture and birch-spruce mixture shared the lowest proportion of OTUs (37.7%, 307 OTUs).

To estimate the magnitude of host misallocation, we assessed colonization of non-host EcM fungi on the root surface of fine root fragments. We compared the relative abundance of the pine-specific *Suillus* species (*S. bovinus*, *S. luteus*, and *S. variegatus*) and birch-specific *Leccinum* species (*L. pulchrum* and *L. variicolor*) on birch, pine and spruce roots. *Suillus* spp. accounted for 3.25% of the sequences recovered from pine roots but 1.03 and 0.07% on birch and spruce, respectively. *Leccinum* spp. formed 0.51% of the sequences connected with birch and 0.04 and 0.00% from spruce and pine, respectively. These specific fungi were recovered from non-host trees only in mixed forest plots. Species of both fungal genera exhibit a long-distance hyphal exploration type and produce high amounts of mycelium (Agerer, 2001), probably indicating the upper limit for incorrect detection.

### Fungal Richness

**Figure 3:**
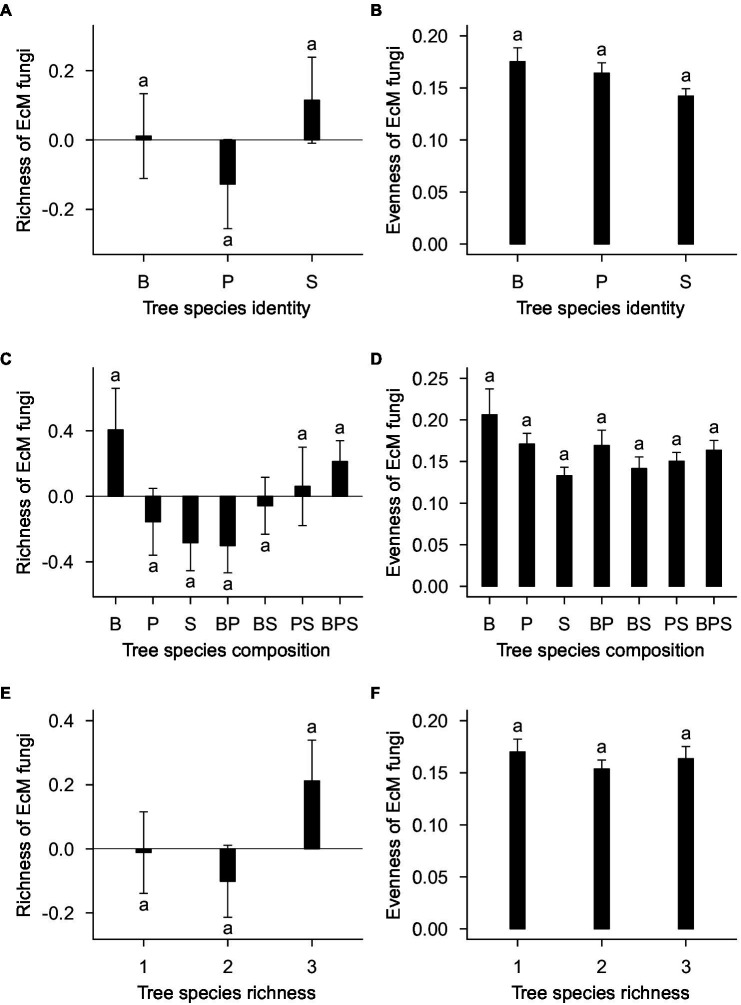
**(A)** Tree species identity, **(C)** tree species composition, and **(E)** tree species richness effects on richness of EcM fungi. **(B)** Tree species identity, **(D)** tree species composition, and **(F)** tree species richness effect on evenness of EcM fungi. EcM fungal richness estimation is based on standardized residuals. Data represent the means of all samples with SEs. Different letters denote significant differences (*p* < 0.05) among factor levels. B, birch; P, pine; S, spruce; BP, birch-pine; BS, birch-spruce; PS, pine-spruce; BPS, birch-pine-spruce; 1, monospecific; 2, two-species mixtures; and 3, three-species mixture.

**Figure 4:**
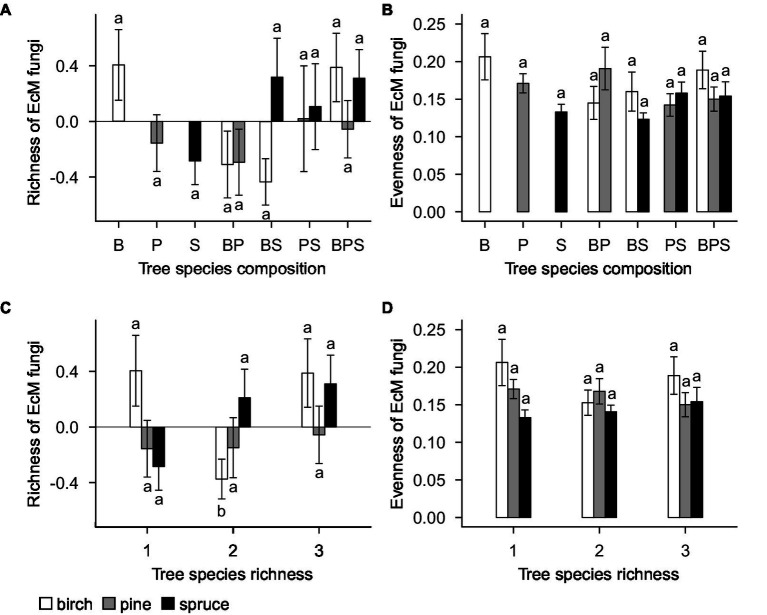
**(A)** Tree species composition and **(C)** tree species richness effects on richness of EcM fungi. **(B)** Tree species composition and **(D)** tree species richness effects on evenness of EcM fungi. EcM fungal richness estimation is based on standardized residuals. Data represent the means of all samples with SEs. Different letters denote significant differences (*p* < 0.05) among factor levels. B, birch; P, pine; S, spruce; BP, birch-pine; BS, birch-spruce; PS, pine-spruce; BPS, birch-pine-spruce; 1, monospecific; 2, two-species mixtures; and 3, three-species mixture.

The standardized residual model of EcM fungal richness was non-significantly affected by tree species identity (*F*_20,143_ = 1.29; *R*^2^_adj_ = 0.019; *p* = 0.196; Figure 3A), tree species composition (*F*_4,143_ = 1.22; *R*^2^_adj_ = 0.013; *p* = 0.326; Figure 3C), plot (*F*_21,143_ = 1.16; *R*^2^_adj_ = 0.044; *p* = 0.371), and tree species richness (*F*_2,143_ = 1.14; *R*^2^_adj_ = 0.006; *p* = 0.345; Figure 3E). Separate standardized residual models for birch, pine, and spruce also showed that there were no significant effects of plot, tree species composition (Figure 4A) and tree species richness (Figure 4C; Supplementary Table S4). However, tree species richness was found to have significant positive impact on EcM richness associated with birch samples (*F*_2,46_ = 4.25; *R*^2^_adj_ = 0.135; *p* = 0.041; Supplementary Table S4).

Tree species identity (*F*_20,143_ = 1.15; *R*^2^_adj_ = 0.003; *p* = 0.306; Figure 3B), tree species composition (*F*_4,143_ = 1.40; *R*^2^_adj_ = 0.023; *p* = 0.262; Figure 3D), plot (*F*_21,143_ = 1.48; *R*^2^_adj_ = 0.065; *p* = 0.194) and tree species richness (*F*_2,143_ = 0.42; *R*^2^_adj_ = −0.003; *p* = 0.662; Figure 3F) had no significant effects on evenness of EcM fungi. When birch, pine, and spruce were analyzed separately, there were no significant effects of tree species composition (Figure 4B), tree species richness (Figure 4D)and plot on evenness of EcM fungi. However, evenness was significantly affected by plot in spruce root samples (*F*_12,49_ = 2.45; *R*^2^_adj_ = 0.200; *p* = 0.014). We summed the number of OTUs separately for each tree species within a plot and compared it between plots of monocultures, two- and three-species mixtures. Tree species richness had non-significant effect on the number of EcM OTUs in birch (*F*_2,13_ = 2.08; *p* = 0.165), pine (*F*_2,13_ = 0.18; *p* = 0.834), and spruce root samples (*F*_2,13_ = 0.60; *p* = 0.564).

### Fungal Community Structure

**Figure 5:**
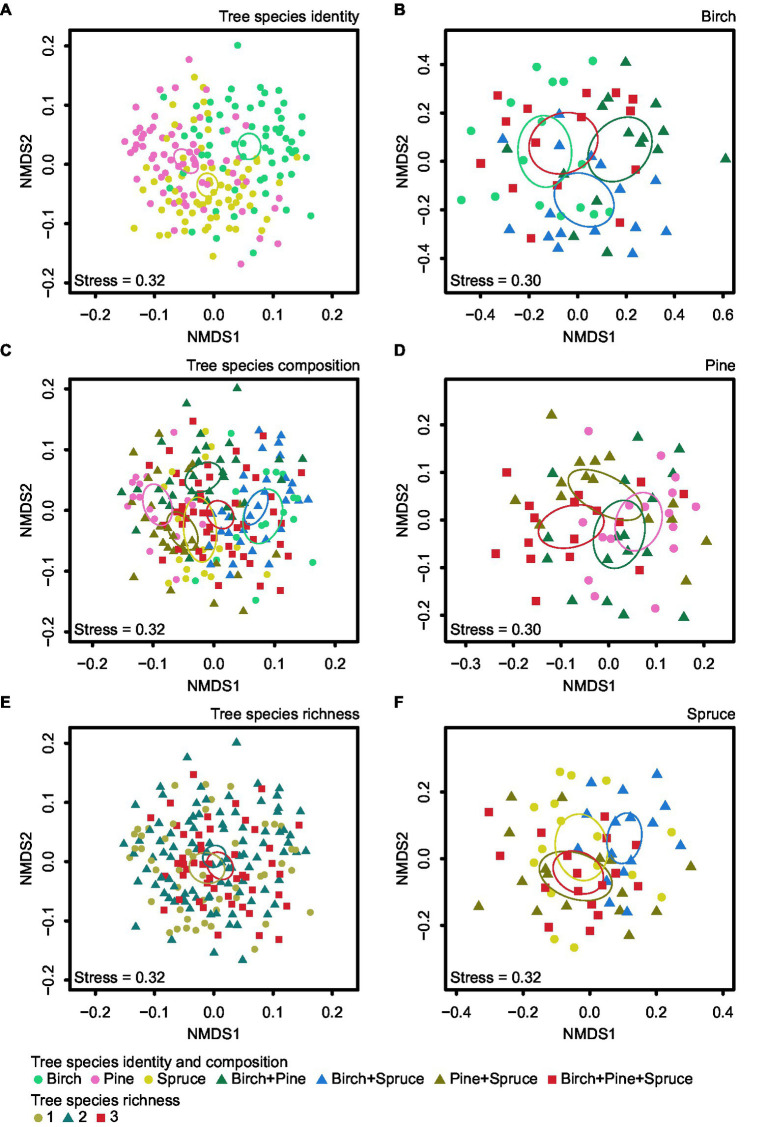
NMDS plots of EcM fungal community composition for the three studied factors: **(A)** tree species identity, **(C)** tree species composition, and **(E)** tree species richness. Tree species composition effects were visualized separately for **(B)** *B. pendula*, **(D)** *P. sylvestris* and **(F)** *P. abies*. Ellipses denote 95% CIs for the different groups.

**Figure 6:**
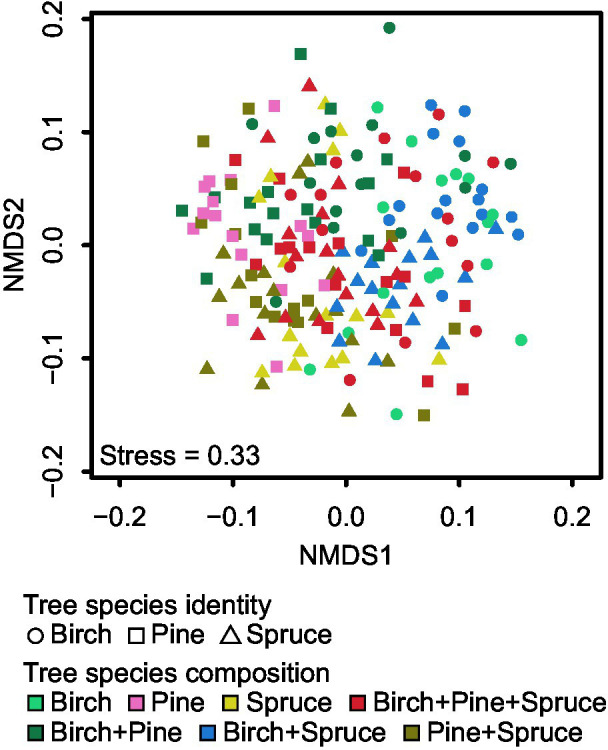
Non-metric multidimensional scaling (NMDS) plots of EcM fungal community composition for tree species identity and tree species composition.

Community structure of all EcM fungi was significantly affected by plot (*F*_pseudo_ = 1.31; *R*^2^_adj_ = 0.057; *p* < 0.001), overall tree species composition (*F*_pseudo_ = 1.85; *R*^2^_adj_ = 0.035; *p* < 0.001; Figure 5C), and tree species identity (*F*_pseudo_ = 1.28; *R*^2^_adj_ = 0.013; *p* < 0.001; Figure 5A; Supplementary Table S5). Tree species richness had no significant effect on community composition of all EcM fungi (*F*_pseudo_ = 0.90; *R*^2^_adj_ = 0.004; *p* = 0.873; Figure 5E; Supplementary Table S5). We tested the effect of tree species identity and tree species composition interaction term on community structure of all EcM fungi. Tree species identity × tree species composition effect on EcM fungal community structure was non-significant (*F*_pseudo_ = 0.95; *R*^2^_adj_ = −0.001; *p* = 0.695; Figure 6).

Plot influenced significantly EcM fungal community composition in birch (*F*_pseudo_ = 1.57; *R*^2^_adj_ = 0.091; *p* < 0.001), pine (*F*_pseudo_ = 1.46; *R*^2^_adj_ = 0.074; *p* < 0.001), and spruce (*F*_pseudo_ = 1.55; *R*^2^_adj_ = 0.085; *p* < 0.001) samples, when these were analyzed separately (Supplementary Table S6). EcM community composition was affected by neighboring tree species composition in spruce root samples (*F*_pseudo_ = 1.55; *R*^2^_adj_ = 0.018; *p* = 0.007; Figure 5F), but not in samples of other trees (*p* > 0.1; Supplementary Table S6).

## Discussion

Our results provide no evidence for a positive relationship between tree species richness and EcM fungal richness on a regional scale in boreal forest stands, which contrasts our first hypothesis. These results are in agreement with previous studies in temperate forests (Nguyen et al., 2016b; Tedersoo et al., 2016) and subtropical forest systems (Chen et al., 2019). However, several studies have found positive tree richness effect on EcM fungal diversity in boreal and temperate forest ecosystems (Kernaghan et al., 2003; Ishida et al., 2007; Tedersoo et al., 2016), indicating the context dependence, i.e., the importance of the study system. Furthermore, on a global scale, EcM tree richness and fungal richness are found to be unrelated (Tedersoo et al., 2012). However, a general soil-based assessment suggested that host plant density and richness, two intercorrelated variables, both had a positive influence on EcM fungal richness (Tedersoo et al., 2014b). On a local scale, other factors, such as tree species identity (Tedersoo et al., 2010; Nguyen et al., 2016b; Nagati et al., 2018; Chen et al., 2019) and phylogenetic distance among hosts (Ishida et al., 2007; Gao et al., 2013; Tedersoo et al., 2013) are shown to have stronger influences on EcM fungal richness than tree species richness *per se*.

In our study, tree species identity had no effect on EcM fungal richness, but it was one of the most important factors affecting EcM fungal composition, which is only partly consistent with the second hypothesis. Host tree species has proven to be the most important factor shaping the community composition of EcM fungi in multiple studies (e.g., Dickie, 2007; Bogar and Kennedy, 2013). Similarly to our results, Nagati et al. (2018) found that dominant tree species affected EcM community composition more than variation of tree species richness. Finding that different tree species tended to host different EcM fungal communities from each other among all plots indicates to some degree of specificity and/or preference in plant-fungi interactions (Santolamazza-Carbone et al., 2019). Host specificity and/or preference are important factors driving EcM community composition (Ishida et al., 2007) that are often displayed at higher plant taxonomic levels among phylogenetically distant hosts (Kernaghan et al., 2003; Põlme et al., 2013; Tedersoo et al., 2013; Molina and Horton, 2015). In our study, the NMDS plots also showed less distance (i.e., higher community similarity) between EcM fungal communities of pine and spruce than between both of the conifers and birch (Figure 5). Furthermore, pine and spruce shared more OTUs than pairwise combinations with birch.

Ectomycorrhizal fungi vary in their host preference and/or specificity. The most common EcM fungi have broad host range, whereas several of the uncommon species are restricted to certain host family or genus (Molina et al., 1992; Molina and Horton, 2015). The level of host specificity in ecosystems strongly depends on the study system and selection of host species (Horton and Bruns, 1998; Ishida et al., 2007; Tedersoo et al., 2008, 2010, 2011; Smith et al., 2009; Peay et al., 2010; Bahram et al., 2014). In our study, Venn diagram showed relatively low proportions of unique OTUs associated with each tree species indicating relatively low host specificity, but we anticipate that the biological pattern of specificity may be severely underestimated because of the recovery of known host-specific fungal taxa, such as *Suillus* spp. (Kretzer et al., 1996) and *Leccinum* spp. (Den Bakker et al., 2004) from non-host tree roots, most likely as rhizoplane or intraradical hyphae (Schneider-Maunoury et al., 2019).

Our third hypothesis predicted that EcM fungal richness and community composition associated with each tree species vary depending on stand species composition. The entire dataset analysis and also separate analysis for birch, pine, and spruce root samples showed that EcM fungal richness in all tree species remained unaffected by tree species composition. However, tree species composition influenced significantly EcM fungal community composition of spruce but not the other trees. The NMDS ordination showed that EcM fungal community structure of spruce differed the most in birch-spruce mixture compared to spruce monoculture (Figure 5F). Similarly, Hubert and Gehring (2008) found no neighboring heterospecific EcM host effect on EcM fungal richness, but showed that while pinyon pine (*Pinus edulis*) EcM community structure was insensitive to the presence of ponderosa pine (*Pinus ponderosa*) neighbors, the EcM fungal community structure of ponderosa pine was affected by the neighboring pinyon pine. In addition, Bahram et al. (2011) reported neighboring tree species effects on mycobiont community composition of *Populus tremula*.

Neighborhood of other host trees can affect EcM fungal communities of focal trees. Competition among EcM fungi of different host trees may have negative effects or facilitation and niche differentiation may have positive effects on overall EcM fungal diversity (Vellend, 2008). Neighboring tree species could exert indirect feedback effects through improved soil conditions (Brandtberg et al., 2000; Légaré et al., 2005). Conifer litter, including spruce (Strack et al., 1989), is rich in phenolic compounds that results in accumulation of recalcitrant organic layer, however, birch-spruce stands with more heterogeneous litter inputs and improved soil physical and chemical properties create opportunities for niche differentiation that may influence EcM fungal communities (Brandtberg et al., 2000; Dickie, 2007; Aponte et al., 2013). Forest stands with the admixture of broad-leaved and coniferous tree species can be associated with higher microbial activity, lower accumulation of organic matter on the forest floor and increase in soil pH resulting in higher total yields and better quality of timber compared with pure stands (Brandtberg et al., 2000; Schua et al., 2015).

## Conclusion

Our results indicate that tree species identity is the most important factor determining EcM fungal community structure in a boreal forest. Compared with the effects of tree species identity, tree species richness had no significant influence on EcM fungal community composition or richness. We found that EcM fungal species richness did not depend on tree species composition and EcM fungal community composition of *Picea abies* (but not that of *Betula pendula* and *Pinus sylvestris*) was modified by the presence of a heterospecific neighbor.

## Data Availability Statement

The sequence data have been released in the Sequence Read Archive (SRA) database (NCBI) under accession number PRJNA534113.

## Author Contributions

LT, JK, and EO designed the study. EA conducted molecular analysis. SA performed bioinformatic analysis. EO annotated metadata, analyzed the data, and wrote the first draft of the manuscript. All authors contributed to the article and approved the submitted version.

### Conflict of Interest

The authors declare that the research was conducted in the absence of any commercial or financial relationships that could be construed as a potential conflict of interest.

## Footnotes

^1^www.fundiveurope.eu
