# Supplementary material for: Tree Species Richness and Neighborhood Effects on Ectomycorrhizal Fungal Richness and Community Structure in Boreal Forest
Source: Front Microbiol. 2021 Feb 22;12:567961. doi: 10.3389/fmicb.2021.567961 (PMC7939122; doi:10.3389/fmicb.2021.567961)
Supplement: Supplementary file 2 [file Data_Sheet_2.docx]

Supplementary Material


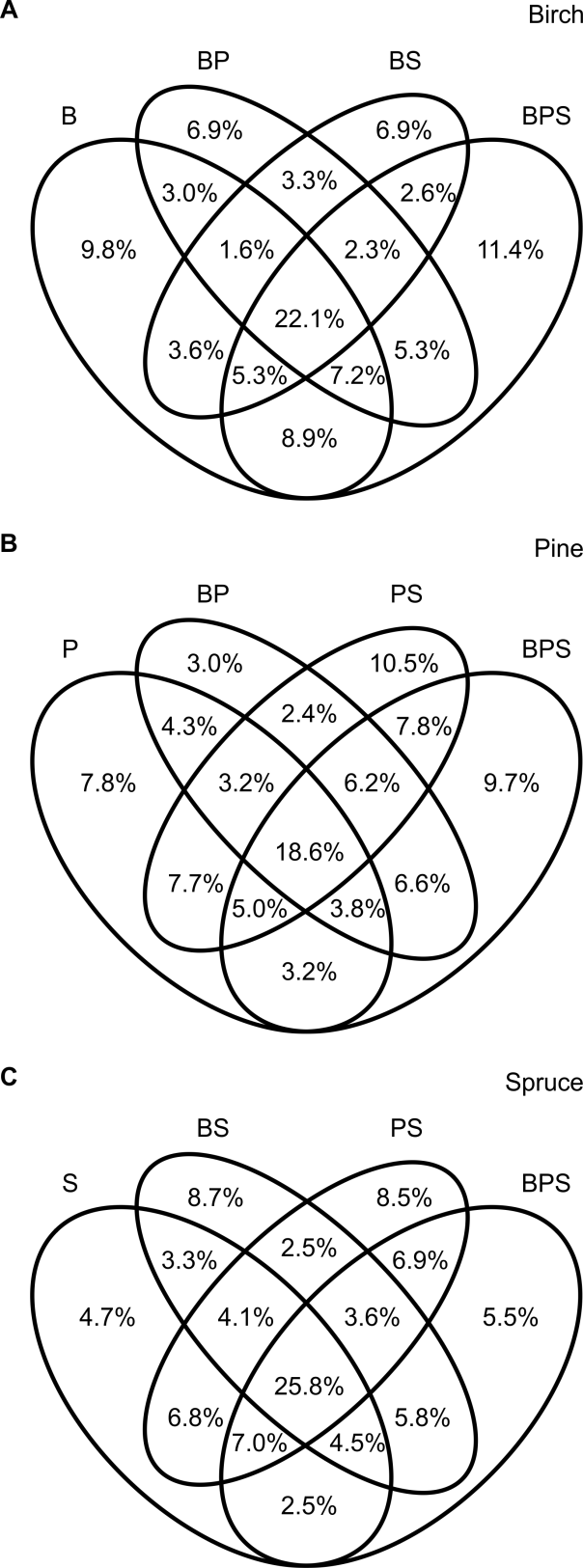


**Supplementary Figure S1.** Venn diagrams displaying the proportion of EcM fungal OTUs of **(A)** *Betula pendula*, **(B)** *Pinus sylvestris* and **(C)** *Picea abies* shared among different forest stands (monoculture and mixed stands). B, birch; P, pine; S, spruce; BP, birch–pine; BS, birch–spruce; PS, pine–spruce; BPS, birch–pine–spruce.

**Supplementary Table S4.** The best predictors for standardized residuals of OTU richness of EcM fungi associated with birch, pine and spruce.

| **Variables** | ***df*** | ***R^2^* _adj_** | ***F*-value** | ***P*-value** |
| --- | --- | --- | --- | --- |
| **Birch** |  |  |  |  |
| Tree richness | 2 | 0.135 | 4.25 | 0.041 |
| Tree composition | 1 | -0.015 | 0.11 | 0.746 |
| Plot | 12 | 0.041 | 1.46 | 0.175 |
| **Pine** |  |  |  |  |
| Tree richness | 2 | -0.031 | 0.03 | 0.967 |
| Tree composition | 1 | -0.005 | 0.47 | 0.508 |
| Plot | 12 | 0.122 | 1.69 | 0.098 |
| **Spruce** |  |  |  |  |
| Tree richness | 2 | 0.023 | 1.68 | 0.228 |
| Tree composition | 1 | -0.010 | 0.36 | 0.562 |
| Plot | 12 | 0.005 | 1.06 | 0.410 |

**Supplementary Table S5.** Multivariate model for community composition of all EcM fungi.

| **Variables** | ***df*** | ***R^2^* _adj_** | **Pseudo-*F*** | ***P*-value** |
| --- | --- | --- | --- | --- |
| Tree richness | 2 | 0.004 | 0.90 | 0.873 |
| Tree composition | 4 | 0.035 | 1.85 | 0.001 |
| Plot | 21 | 0.057 | 1.31 | 0.001 |
| Tree identity (Plot) | 20 | 0.013 | 1.28 | 0.001 |

**Supplementary Table S6.** Multivariate models for community composition of EcM fungi associated with birch, pine and spruce.

| **Variables** | ***df*** | ***R^2^* _adj_** | **Pseudo-*F*** | ***P*-value** |
| --- | --- | --- | --- | --- |
| **Birch** |  |  |  |  |
| Tree richness | 2 | 0.011 | 0.96 | 0.573 |
| Tree composition | 1 | 0.014 | 1.34 | 0.073 |
| Plot | 12 | 0.091 | 1.57 | 0.001 |
| **Pine** |  |  |  |  |
| Tree richness | 2 | 0.010 | 1.00 | 0.491 |
| Tree composition | 1 | 0.009 | 1.16 | 0.200 |
| Plot | 12 | 0.074 | 1.46 | 0.001 |
| **Spruce** |  |  |  |  |
| Tree richness | 2 | 0.005 | 0.84 | 0.911 |
| Tree composition | 1 | 0.018 | 1.55 | 0.007 |
| Plot | 12 | 0.085 | 1.55 | 0.001 |
